# Microbial enzyme secretion: An annotated selection of World Wide Web sites relevant to the topics in *microbial biotechnology*.

**DOI:** 10.1111/1751-7915.12702

**Published:** 2017-02-27

**Authors:** Lawrence P. Wackett

**Affiliations:** ^1^ Department of Biochemistry, Molecular Biology and BiophysicsBioTechnology InstituteUniversity of MinnesotaSt PaulMN55108USA

## Fungal secretome knowledge base

### http://bioinformatics.ysu.edu/secretomes/fungi.php

The Fungal Secretome KnowledgeBase contains data from many fungi. One can look for secreted proteins or input a particular protein to see whether it is secreted.

## Database of lignocellulose‐degrading enzymes from fungi

### https://mycoclap.fungalgenomics.ca/mycoCLAP/

This database focuses on fungal, and some bacterial, enzymes that have activity against lignocellulose materials. Many of those enzymes are secreted from their respective microbial host.

## Peptidase database

### http://merops.sanger.ac.uk/cgi-bin/name_index?id=P;action=A

This database focuses on protease and peptidase enzymes. Many microbes living in soil environments secrete proteases to break down proteins into amino acids and peptides that can be transported into the cell.

## Database for type IV secretion system

### http://www.t4ss.lncc.br

This small database is focused on bacterial type IV secretion systems.

## Identification of secretion systems in bacterial genomes

### http://www.nature.com/articles/srep23080

This relatively recent publication deals with computational methods for identifying secretion system genes in genomic sequences.

## Bacterial secretion systems review

### https://www.ncbi.nlm.nih.gov/pmc/articles/PMC4804464/

This review is on bacterial secretion systems with a major focus on their role in promoting bacterial virulence.

## Type three secretion system

### https://en.wikipedia.org/wiki/Type_three_secretion_system

This Wikipedia entry provides a fairly comprehensive overview of bacterial type III secretion systems.

## X‐ray structure of a type III secretion system component

### https://www.ncbi.nlm.nih.gov/pmc/articles/PMC4400350/

This paper deals with the structure of a protein component that aids the process by which type III secretion systems traverse the peptidoglycan layer.

## Killing via a type IV secretion system

### http://www.nature.com/articles/ncomms7453

This study shows that the type IV secretion system of *Xanthomonas citri* can kill other Gram‐negative bacteria in a contact‐dependent manner.

## OmpA‐facilitated chitinase secretion

### https://springerplus.springeropen.com/articles/10.1186/s40064-016-2893-y

Protein modification with a OmpA signal peptide was most productive with respect to secretion of a *Bacillus* chitinase by *E. coli*.

## *Streptomyces* protein secretion biotechnology

### http://www.sciencedirect.com/science/article/pii/S0167488914000093

Many *Bacillus* and *Streptomyces* strains are good protein secretors. This study demonstrated *Streptomyces lividans* to be a particularly good secretion host.

## Optimization of protein secretion by *Bacillus subtilis*

### https://www.ncbi.nlm.nih.gov/pubmed/19075856

This article deals mostly with commercially important aspects of *Bacillus* secretion systems and reviews recent patents in the area.

## Phytase secretion by *Bacillus subtilis*

### http://www.tandfonline.com/doi/pdf/10.1080/09168451.2015.1046366

Phytases are industrially important enzymes for their ability to release phosphate from phytic acid. This study explores its secretion by a *Bacillus* strain.

## Secretion of a thermostable amylase

### http://www.nature.com/articles/srep22229

An amylase from *Pyrococcus furiosus* is significant industrially because of its stability at high temperatures and low pH. It is shown here to be secreted by a *Bacillus amyloliquefaciens* strain.

## Protein secretion by *Pichia pastoris* review

### https://www.ncbi.nlm.nih.gov/pmc/articles/PMC4047484/

*Pichia* is a methylotrophic yeast that grows to high cell density, and this review offers ideas for optimizing protein secretion by this strain.

## Methyl parathion hydrolase secretion by *Pichia*

### http://journals.plos.org/plosone/article?id=10.1371/journal.pone.0096974

This paper deals with the secretion of an enzyme for biodegrading a common pesticide.

## *Pichia* technology

### http://www.pichia.com

This commercial site contains significant and useful information on *Pichia* and its secretion systems.

## Pichia secretion tools

### https://www.thermofisher.com/order/catalog/product/A11155

This commercial page has lnks to information on *Pichia*.

## Fungal biomass degrading enzymes from *Pichia*

### http://journal.frontiersin.org/article/10.3389/fmicb.2015.01002/full

The review details the use of *Pichia* for producing carbohydrate‐hydrolysing enzymes that are highly useful in biodegrading biomass.

## Patent on protease deficient *Pichia* secretion strain

### https://www.google.com/patents/US8703444

Naturally produced proteins can cause problems in high‐level expression of heterologous proteins, and this patent describes a protease deficient mutant that aids expression of intact protein.

## Yeast secretion strains

### https://www.atum.bio/eCommerce/catalog/datasheet/243

This commercial website contains much useful information on the construction and use of *Pichia* expression and secretion systems.

## High‐level protein production in *Streptomyces lividans*

### https://microbialcellfactories.biomedcentral.com/articles/10.1186/s12934-016-0425-7

An amylase, laccase and xylanase were used as reporters to optimize secreted expression in *Streptomyces lividans*.

## Development of a *Corynebacterium glutamicum* secretion system

### http://onlinelibrary.wiley.com/doi/10.1002/bit.25692/abstract

*Corynebacterium glutamicum* is a very important industrial strain for amino acid production. This study investigated establishing this microbe for protein secretion.

## *Aspergillus* protein secretion

### http://bmcsystbiol.biomedcentral.com/articles/10.1186/1752-0509-8-73

*Aspergillus oryzae* is an important strain for secreting industrial enzymes, and it also performs necessary post‐translational modifications. This study was conducted to better understand the fundamental molecular machinery involved.

